# Case report: *Anaerobiospirillum* prosthetic joint infection in a heart transplant recipient

**DOI:** 10.1186/s12891-019-2684-z

**Published:** 2019-06-26

**Authors:** Gregory R. Madden, Melinda D. Poulter, Michael P. Crawford, Daniel S. Wilson, Gerald R. Donowitz

**Affiliations:** 1 Division of Infectious Diseases & International Health, Department of Medicine, University of Virginia Health System, P.O. Box 800473, Charlottesville, VA 22908-0473 USA; 2 Clinical Microbiology Laboratory. Department of Pathology, University of Virginia Health System, Charlottesville, VA USA

**Keywords:** *Anaerobiospirillum*, Prosthetic joint infection, Solid-organ transplant

## Abstract

**Background:**

We report a case of prosthetic hip joint infection in a heart transplant recipient due to *Anaerobiospirillum succiniciproducens,* a genus of spiral-shaped curved anaerobic gram-negative rod which colonizes the gastrointestinal tract of cats and dogs. Invasive infections in humans are rare and typically occur in immunocompromised hosts.

**Case presentation:**

A 65-year-old male dog breeder with a history of rheumatoid arthritis, bilateral hip arthroplasties, and non-ischemic cardiomyopathy with a heart transplant 10 years ago presented with a three month history of progressive left hip pain and frank purulence on hip aspiration. He underwent irrigation and debridement of the left hip and one-stage revision with hardware exchange. Although gram stain and culture from synovial fluid and intraoperative cultures were initially negative, anaerobic cultures from tissue specimens later grew a spiral-shaped gram-negative rod, identified as *Anaerobiospirillum* spp. by 16S rRNA gene sequencing. The patient was treated with ceftriaxone 2 g daily for 6 weeks with a good response to treatment. A similar organism was unable to be isolated from culture of 2 of the patient’s dogs, however, they were thought to be the most likely source of his infection.

**Conclusion:**

*Anaerobiospirillum spp*. should be considered in immunocompromised patients with exposure to dogs or cats who present with bacteremia, gastrointestinal infection, pyomyositis, or prosthetic joint infections, especially in cases of culture-negative or with anaerobic culture growth.

## Background

*Anaerobiospirillum* are spiral-shaped anaerobic gram-negative rods that were first isolated in 1976 from the throats and feces of beagles. *A. succiniciproducens* is thought to be part of normal gastrointestinal flora of some dogs and cats [1], but not humans [2]. Infections due to *Anaerobiospirillum* are rare but instances of bacteremia, pyomyositis, gastrointestinal infections, and one case of knee prosthetic joint infection (PJI) has been described [2–5]. These infections typically in immunocompromised hosts or patients with comorbid medical conditions.

## Case presentation

A 65-year-old male presented with a three month history of progressive left hip pain. His past medical history was noteworthy for a non-ischemic cardiomyopathy for which he underwent a heart transplant ten years prior to presentation. He had a history of osteoarthritis for which he had undergone bilateral hip replacements complicated by a left prosthetic hip infection with *Cutibacterium acnes.* His past history was also positive for rheumatoid arthritis, hypertension, and type 2 diabetes.

The first stage of his left hip revision after his first episode of prosthetic joint infection with *C. acnes* occurred pre-heart transplantation and he was treated with 6 weeks of vancomycin following hardware explantation. His second-stage hip revision occurred over a year later, following his heart transplant. His post-transplant course was otherwise uncomplicated, without any episodes of graft dysfunction, rejection, or infectious complications over the previous nine years. He was maintained on tacrolimus and mycophenolate mofetil since his transplant without recent corticosteroid exposure.

With regard to his present symptoms, along with the pain, he noted occasional night sweats. He reported receiving 2 hip injections for the pain (one into his hip joint and another into his trochanteric bursa, both on his left side) by his outpatient orthopedic doctor about 2 weeks prior to presentation that provided temporary relief. Sterile precautions were reportedly observed during in-clinic procedures. Following the injections, the patient noticed a progressive fluctuant area swelling of his left lateral thigh and hip. Aspiration of that area 4 days prior by his outpatient orthopedist yielded purulent-appearing fluid that was Gram stain negative for organisms, with no growth seen either on aerobic or anaerobic cultures. Three days prior to admission, he underwent hip arthrocentesis that showed cloudy fluid with 65,000 white blood cells per microliter and a differential of 95% polymorphonuclear neutrophils. Gram stain was negative, and no crystals were seen. Culture of the synovial fluid was later finalized as no growth. However, prosthetic joint infection was suspected and so the patient was admitted for surgery.

The patient was an active dog breeder (Yorkies, Maltese, and Morkies), with over 10 dogs at home at any one time, and had recently participated in the birth of several puppies. There was no history of illicit or injection drug use. He also noted being scratched on his legs by the dogs in the past, but could not recall any recent scratches or bites recently.

On admission, he was well appearing and comfortable at rest. All vital signs were normal and he was afebrile. He noted mild pain with passive and active movement of his left hip, however, he was able to ambulate. Oropharyngeal exam was unremarkable with good dentition. His bilateral shins had evidence of previously-healed scratches. On the lateral aspect of his thigh was a tender egg-sized swollen area of fluctuance and erythema, without increased warmth or obvious drainage or sinus tract.

Laboratory evaluation was notable for a peripheral white blood cell count of 6300 per microliter. Erythrocyte sedimentation rate and C-reactive protein levels were 63 mm/hour and 5.4 mg/dL, respectively. Hip X-ray did not show loosening or disruption of his prosthesis. He did not receive any antibiotics prior to surgery. Two sets of pre-operative blood cultures were drawn and finalized as no growth. Medications at the time of presentation included methotrexate (2.5 mg daily), mycophenolate mofetil (250 mg twice daily), and tacrolimus (1 mg twice daily). His diabetes mellitus was well-controlled with dietary changes and his most recent hemoglobin A1c measurement was 6.2%.

The area of the suspected abscess the lateral thigh was incised and drained. The proximal prosthetic body, femoral head, and acetabular liner were all removed, the joint was irrigated and debrided, and hardware was replaced in a one-stage fashion. Vancomycin and tobramycin-containing beads were placed. Intraoperatively, frank purulence within the joint was not observed. Five separate tissue specimens were sent for bacterial, fungal, and mycobacterial culture. Post-operatively, vancomycin and ceftriaxone were begun empirically pending further culture data.

**Fig. 1:**
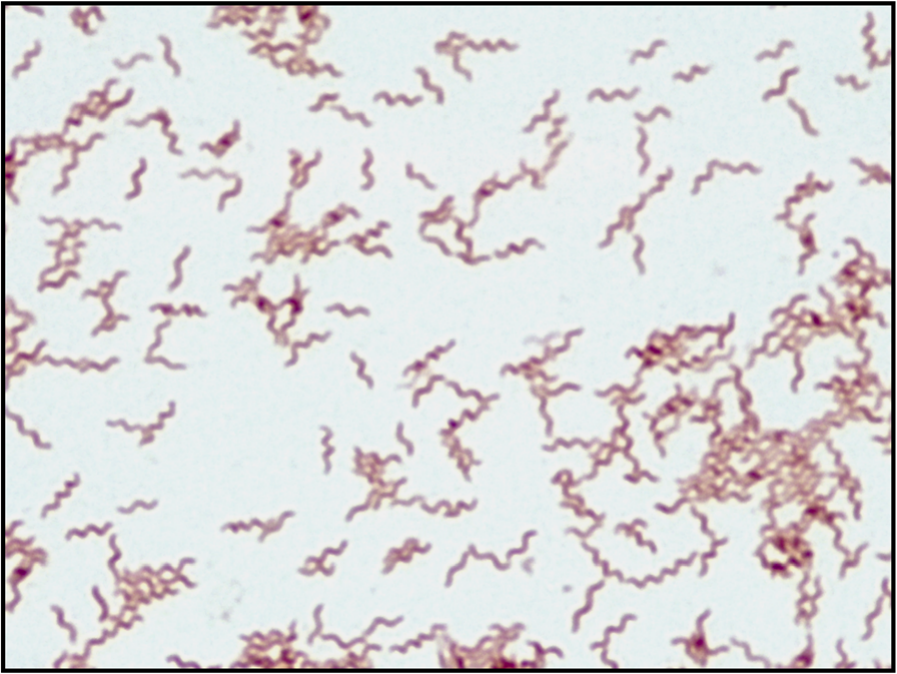
Gram stain from solid media demonstrated spiral-shaped gram-negative rods (1000x magnification, with oil)

Approximately 2-3 mm of each tissue submitted was embedded whole in fungal media. The remaining tissue from each site was ground in 1 mL Tryptic Soy Broth. Mycobacterial, fungal, and bacterial growth media were inoculated with ground tissue suspension. Gram, Acid Fast, and Calcofluor White stains were prepared from touch preps of intact tissue and ground tissue suspensions. All stains of tissue were negative for organisms. Bacterial cultures were incubated at 35 °C in a 5–10% CO_2_ incubator for aerobic cultures and in the BD BBL GasPak anaerobic pouch for anaerobic cultures. After four days of incubation, tissue cultures collected from the left hip (one of 5 total submitted) grew small, translucent, spreading colonies from the anaerobic blood agar incubated under anaerobic conditions. Gram stain of the colonies demonstrated spiral-shaped gram-negative rods (Fig. 1). Matrix-assisted laser desorption time of flight (MALDI-TOF) mass spectrometry yielded and identification of *Anaerobiospirillum succiniciproducens*, which is currently unclaimed in the bioMerieux Vitek-MS database. Therefore, 16 s ribosomal RNA gene sequencing was performed, with the best matches using NCBI Blast Nucleotide Sequence Database to *Anaerobiospirillum succiniciproduces* and *Anaerobiospirillum* species with > 99% query coverage and identity. Ultimately, the laboratory report was released as “most closely resembles *Anaerobiospirillum* species; Identified by DNA sequencing.” 16S sequencing was not performed on any other samples including blood.

Mycophenolate was held temporarily in the setting of infection. Following identification of the organism, vancomycin was discontinued and ceftriaxone (2 g daily) was continued to complete 6 weeks total of antibiotic therapy. He tolerated treatment well to date, without evidence of recurrent infection.

Saliva and perirectal anaerobic cultures of two of the patient’s dogs (both Maltese) were performed using flocked swabs submitted in anaerobic agar gel transport media, and cultured on standard anaerobic media. No organisms resembling *Anaerobiospirillum spp.* were isolated.

Follow up 2 months later was unremarkable for relapse of infection, after completion of intravenous antibiotics,.

## Discussion and conclusions

*Anaerobiospirillum* infections are rare and this is the second reported case of this organism causing PJI, the first involving a hip prosthesis, and the first joint infection related to orthopedic injection [3].

Most *Anaerobiospirillum* infections involving sepsis or invasive infections (i.e., bacteremia and pyomyositis) occur in immunocompromised or patients with significant comorbidities. In one series of patients with bloodstream infections due to *Anaerobiospirillum succiniciproducens*, over 90% had significant comorbid conditions (including over a third with alcohol abuse) or immune-suppression [4]. Diarrheal infections occur in healthy patients [4, 6] and one case of bacteremia was reported in a healthy man [2]. Mortality rate of infections due to *Anaerobiospirillum* approximate 30% [5].

Another *Anaerobiospirillum species, A. thomasii,* has also been identified but less is known about this organism. It has been isolated from both diarrheal specimens from humans and from asymptomatic cats and dogs [6].

*Anaerobiospirillum* are thought to be acquired via zoonotic transmission. Dog ownership is a well described risk factor for *Anaerobiospirillum* infection and in one study, *A. succiniciproducens* was isolated from the GI tract of 8% of healthy dogs [7]. While there is indirect evidence to suggest zoonotic transmission of *Anaerobiospirillum* from cats and dogs to humans, such as cat bites [8] or exposure to excrement [9], this microepidemiologic link is not well established. In one series of 24 patients with *A. succiniciproducens* bloodstream infection, only 3 had a documented exposure to animals [4] and an odontogenic source was suspected in some cases [6, 7]. There have been two reports of a morphologically similar organism cultured from the pet dogs of patients with *Anaerobiospirillum* gastrointestinal infections [10, 11], but no such epidemiologic link has been described in cases of invasive infections. Human-to-human transmission has not been reported.

Although the patient’s dogs were the suspected primary source, *Anaerobiospirillum* was not able to be isolated from the animals. However, culture of this organism may be difficult and only a fraction of the patient’s many dogs were cultured. We suspect the route of infection was most likely local introduction from the skin via his recent hip injections. An alternative explanation could have been hematogenous dissemination from scratches on the patient’s legs. There was no evidence of pyomyositis on imaging and no fistulas were detected. Dissemination from an odontogenic source or gastroenteritis were also possible. However, the patient’s dentition was good and he denied recent gastrointestinal symptoms.

Septic arthritis is a rare complication of joint aspiration or injection, occurring in approximately 0.04% of procedures [12]. Although the exact mechanism is unclear, injection-related infections may occur through inoculation of skin flora by tissue coring or (in rarer circumstances) due to contamination of the injectate [13]. Repeated injections is also an identified risk factor for PJI [14].

The optimal antibiotic treatment for *Anaerobiospirillum* infections is unknown. Somewhat unique among anaerobic bacteria, in vitro metronidazole resistance has been reported (75% of isolates), as well as resistance to clindamycin (90% of isolates) [4]. Beta lactams generally have good activity against *A. succiniciproducens*, and successful treatment has been described with penicillin [15, 16], aminopenicillins [17, 18], and cephalosporins [4, 19]. Treatment with quinolones, aminoglycosides, chloramphenicol, and tetracyclines are reported as well [4, 14].

*Anaerobiospirillum spp.* are difficult to culture. A selective fastidious aneaerobe agar medium is described for isolation from stool using polymyxin, vancomycin, and sulfamethoxazole [2]. Once isolated, MALDI-TOF mass spectrometry has been proposed as a method which could help to more rapidly and accurately identify the organism [6, 11]. Wet mount of the bacterium may demonstrate corkscrew motility [9].

Infection with spiral-shaped bacteria is unusual in humans; the differential diagnosis is generally limited to certain genera of spirochetes (*Borrelia, Leptospira, Treponema spp.*) or curved rods (*Helicobacter, Campylobacter spp.*). Accurate identification of infections due to *Anaerobiospirillum* is important for both epidemiologic and treatment purposes. *Anaerobiospirillum* has been mistaken for its related genus, *Campylobacter* [7] and, importantly, *Anaerobiospirillum* are resistant to the antibiotics of choice for *Campylobacter* infection (macrolides). *Anaerobiospirillum* should be considered in cases of culture negative or anaerobic infections in immunocompromised hosts with exposure to cats or dogs.

## Data Availability

Data sharing is not applicable to this article as no datasets were generated or analyzed during the current study. All data contained within the article.

## Abbreviations

MALDI-TOF: Matrix-assisted laser desorption time of flight
PJI: Prosthetic Joint Infection
